# Tumor-Infiltrating Immune Cell Signature Predicts the Prognosis and Chemosensitivity of Patients With Pancreatic Ductal Adenocarcinoma

**DOI:** 10.3389/fonc.2020.557638

**Published:** 2020-09-25

**Authors:** Yuzhen Gao, Shipeng Chen, Somayeh Vafaei, Xiaoli Zhong

**Affiliations:** ^1^Department of Molecular Diagnosis, Clinical Medical College, Yangzhou University, Yangzhou, China; ^2^Department of Laboratory Medicine, Shanghai Eastern Hepatobiliary Surgery Hospital, Shanghai, China; ^3^Department of Molecular Medicine, Faculty of Advanced Technologies in Medicine, Iran University of Medical Sciences, Tehran, Iran

**Keywords:** pancreatic ductal adenocarcinoma, immune cell, prognosis, chemotherapy response, signature

## Abstract

**Objective:**

Tumor-infiltrating immune cells might add a predictive value for the prognostic stratification of patients with pancreatic ductal adenocarcinoma (PDAC) and chemotherapy response. We aimed to develop a prognostic model based on the tumor-infiltrating immune cell signature to improve the prediction of survival and chemotherapy benefits of patients with PDAC.

**Methods:**

The abundance of tumor-infiltrating immune cells for 661 patients with PDAC from four different cohorts with survival data was collected in the training cohorts. Cox regression analysis and meta-analysis of immune cells were conducted to generate the tumor immune cell score (TICS) for prognostic stratification. Other two independent cohorts including 188 patients were then used to validate the model. Those patients who underwent chemotherapy were used to further analyze the value of TICS for predicting the chemotherapy response. Furthermore, the difference in the somatic mutations and immune-related molecules between the TICS subgroups was analyzed.

**Results:**

6 out of 28 immune cells were found to be significantly associated with PDAC prognosis in the training cohorts (all *P* < 0.05). The developed TICS could significantly predict the PDAC survival and chemotherapy benefit both in the training and the external validation cohorts (log-rank test, *P* < 0.05). Significant differences were found in different TICS subgroups in terms of the immune characteristics, checkpoint genes, and tumor mutational burden. Functional and pathway analyses further proved that the TICS was significantly related to the tumor immunity response in patients with PDAC.

**Conclusion:**

TICS might be used to predict PDAC patients with a better survival and greater chemotherapy benefit.

## Introduction

Pancreatic ductal adenocarcinoma (PDAC) is the 4th leading cause of cancer-related deaths worldwide and it consists of almost 90% pancreatic cancers (1, 2). Although in recent years significant improvements have been achieved in the diagnosis and treatment of different kinds of cancers (3), the mortality rate of PDAC has not experienced substantial changes during the past decades. This is because PDAC is a highly destructive cancer with an extraordinarily high malignancy and a particularly poor prognosis (4). Once this aggressive cancer is diagnosed, the 5-year survival rate is only around 7%, and the 1-year survival rate is <20% (5). Surgery is the first option for the resected stage of PDAC and adjuvant chemotherapy is the standard treatment following surgery. Numerous studies have shown that patients’ long-term survival improved after the treatment with adjuvant chemotherapy (6). However, given its poor prognosis among cancer and despite combining the advances in surgical techniques and other adjuvant therapy in recent years, the tumor recurrence rate remains at 80% (6). Currently, the prognostic stratification of a resected PDAC is based on the 8th edition of the AJCC–TNM classification (7), which mostly relies on the tumor invasion parameters containing tumor burden (T), the influence of cancer cells in lymph nodes (N), and presence of metastases (M). However, the differences persist in the individual tumor biology and immune cell characteristics among patients suggesting the weakness of the current prognostic system (8). Thus, the effective and accurate stratification of patients with PDAC for prognosis and chemotherapy response is of great importance.

Although aggressive PDAC renders the development of novel prognosis prediction challenges in current oncological research (9), immune cells within the tumor microenvironment (TME) contribute to the prognostic assessment in a wide range of malignancies (10–12). Highly infiltrated T-lymphocytes, especially CD8^+^ T cells in TME, are associated with a good overall survival (OS) and disease-free survival (DFS). By contrast, patients with a large amount of immune-suppressive cells, such as myeloid-derived suppressor cells (MDSCs), M2 macrophages, and CD4^+^CD25^+^Fop3^+^ Tregs, show a relatively poor outcome because of the block of the cytotoxic T-cell function in the recognition and clearance of cancer cells (13–15). Based on the effect of the tumor-infiltrating immune cells on patients’ prognosis (16), we then questioned whether the features of immune cells benefit the prediction of prognosis and chemotherapy response of PDAC. Thus, the current study aimed to analyze the tumor-infiltrating immune cells to establish a tumor immune cell score (TICS) in order to enhance the prediction of prognosis and chemotherapy response of patients with PDAC. To the best of our knowledge, this study is the first to focus on predicting the survival and chemotherapy treatment responses of patients with PDAC based on the tumor-infiltrating immune cell signatures.

A total of 849 PDAC patients with a detailed medical information were collected from The Cancer Genome Atlas program (TCGA), International Cancer Genome Consortium (ICGC), and Gene Expression Omnibus (GEO) databases. Bioinformatics analysis–based immune cell-specific signatures were applied to characterize the composition of tumor-infiltrating immune cells and immune-related molecules (17). To avoid bias, the prognostic immune cell signatures were selected based on a meta-analysis from the four different clinical cohorts (18). TICS was developed systematically for predicting the survival and treatment response from adjuvant chemotherapy by taking advantage of the tumor-infiltrating immune cell signatures. The significant differences in the clinical characteristics, immune molecules, and tumor mutational burden (TMB) between the high/low TICS subgroups were observed. Furthermore, these results were validated in the two external patient cohorts. High TICS can predict PDAC patients with better survival outcomes and classify patients who will potentially get a greater benefit from an adjuvant chemotherapy.

## Materials and Methods

### Data Preprocessing

All the PDAC transcriptome and clinical data were obtained from the TCGA, ICGC, and GEO databases. Four different cohorts including the TCGA-PAAD-US (*n* = 146), ICGC-PACA-AU (*n* = 267), ICGC-PACA-CA (*n* = 182), and GSE62452 (*n* = 66) were enrolled as the training cohorts. Two other independent cohorts: GSE71729 (*n* = 125) and GSE57495 (*n* = 63), were utilized as the external validation cohorts (Supplementary Table S1). The corresponding somatic mutation data of the enrolled patients were also downloaded from the databases above. All the expression data were normalized, the raw files were applied in the Robust Multi-array Average (RMA) algorithm, and the background adjustment using the “affy” and “simpleaffy” software package. Besides, log2 transform was applied in this analysis. The pan-cancer cohorts from the GDC Pan-Cancer in the UCSC Public Hub were also downloaded for further analysis in the present study^1^.

### Calculation of Tumor-Infiltrating Immune Cells, Tumor Mutation Burden, Immune Score, and Cytolytic Activity Score

The single sample gene set enrichment analysis (ssGSEA) algorithm was used to quantify the relative abundance of the tumor-infiltrating immune cells of patients with PDAC. Seven hundred eighty-two (782) immune genes were obtained based on the previous research (18). These associated genes could be assessed via the package of the “GSVA” in Bioconductor before the treatment schedule. TMB was defined as the number of somatic mutations in the coding region per megabase including the single nucleotide variants (SNVs) and small insertions and deletions (Indels). ImmuneScore was developed by using the “ESTIMATE” packages in each patient (19). Furthermore, the cytolytic activity (CYT) score as the geometric mean of granzymes A (GZMA) and perforin (PRF1) mRNA expression levels in tumor tissue CYT score = $\sqrt{G\,Z\,M\,A\times P\,R\,F\,1}$ (20).

### Generation of TICS

Cox proportional hazards regression analysis was conducted to explore the prognostic evaluation of 28 immune cells. Each immune cell was stratified by the median value into two groups in the four PDAC training cohorts. The pooled hazard ratio (HR) with a 95% CI of each immune cell was estimated by using the fixed-effects model based on meta-analysis. With the immune cells, the pooled HRs with their standard estimates (SE), which were significantly related to prognosis, were then integrated as the prognostic immune cell weight and generated the TICS. In sum, TICS is expressed as follows:

$$\text{TICS}=\sum_{i=1}^{n}\frac{1-\text{HRi}}{\mathrm{SE}\,\left(\mathrm{HRi}\right)}\times G\,S\,V\,A\,\left(C\,e\,l\,l\right)$$

where GSVA (cell) is the relative infiltration of the OS-related immune cells by GSVA and n is the number of the OS-related immune cells based on the meta-analysis. In our analyses, the normalized *Z*-score was used to calculate the score. The best cut-off values of TICS in the different cohorts were truncated by the “survminer” package in the R software.

### Functional and Pathway Enrichment Analysis

The present study also aimed to investigate the potential difference in the biological function between the different TICS subgroups. A total of 96 immune-related gene sets of functional annotations were downloaded from the “msigdb.v7.0.symbols” in the MSigDB. The up- and down-regulated immune-related terms between the different TICS subgroups were identified by running the “pathifier” R package in the TCGA transcripts. Gene Ontology (GO) and Kyoto Encyclopedia of Genes and Genomes (KEGG) functional annotations using the “clusterProfiler” R package were performed based on the alternating TICS signature genes. Enrichment *P*-values were based on 1,000 permutations and subsequently adjusted for multiple testing using the Benjamini–Hochberg procedure to control the FDR.

### Statistical Analysis

Statistical analysis of all the clinical data was performed in R 3.6.2 – standard tests including the Student’s *t*-test, Wilcoxon rank-sum test, and Fisher exact test. The method of Benjamini–Hochberg, which is the same as the FDR in R, was also used to adjust the *P*-values for multiple comparisons. The relationship between TICS and other continuous variables was calculated by the Spearman method. The optimal cutoff values of TICS and TMB were selected by using the “survminer” R package. The log-rank test and univariate and multivariate cox proportional hazard regression was used to explore the related independent predictors of the prognosis. Additionally, time-ROC was used to detect the prognostic value of TICS and other variables for the prognosis of PDAC patients. The area under the receiver operating characteristic curve (ROC) was used to detect the diagnostic value of TICS for chemosensitivity. All reported *P*-values were two-sided, and the statistical significance was set at 0.05.

## Results

### Large-Scale Meta-Analysis Reveals the Prognostic Value of Tumor-Infiltrating Immune Cells in PDAC

**FIGURE 1:**
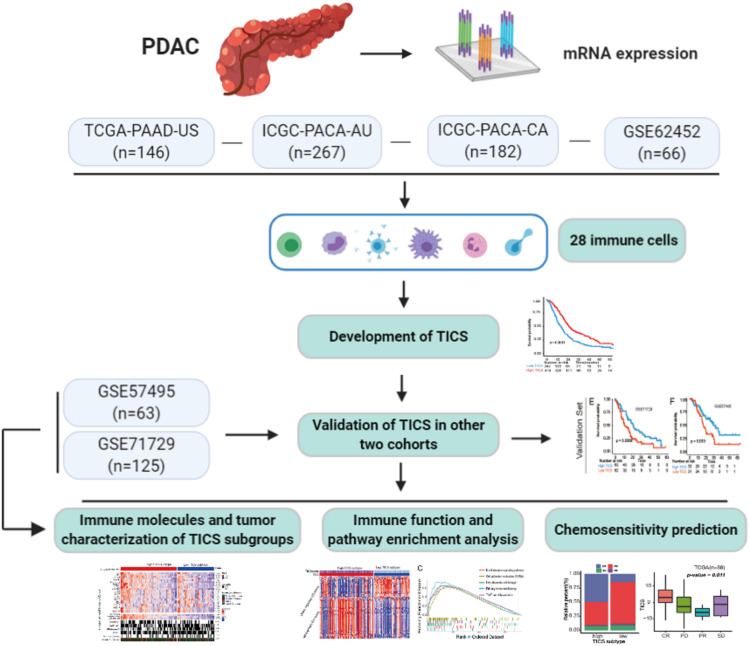
A schematic of the study design and model development. Details of the study design and PDAC patients cohorts classification for model training and validation in regards to the overall survival, immune and tumor characterization, as well as the chemosensitivity prediction.

**FIGURE 2:**
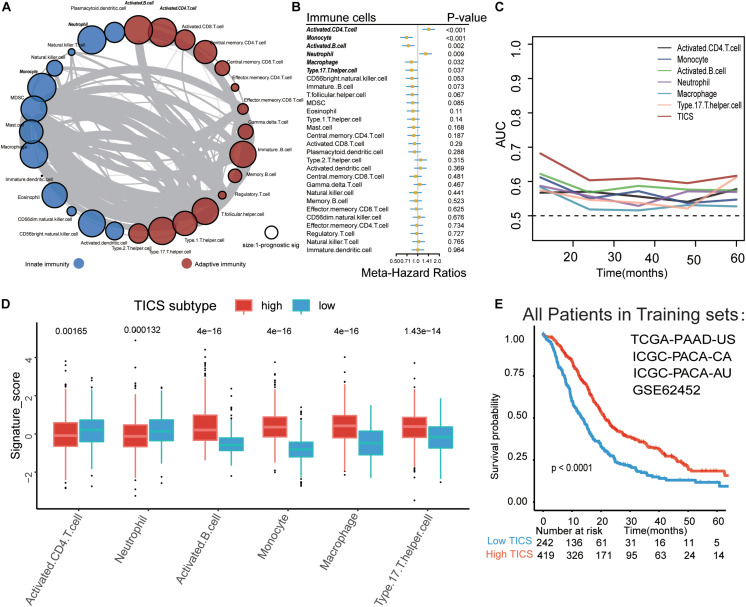
Establishment of Tumor-infiltrating immune cell score (TICS). **(A)** The spearman relationship within the 28 immune cells. **(B)** Forest plot for the prognostic values of the 28 immune cells in PDAC patients; **(C)** Time-dependent receiver operating characteristic curve (ROC) analysis of the six prognostic immune cells and TICS. **(D)** The differences of the six prognostic immune cells between the high and low TICS subgroups which stratified by the best cut-off value. **(E)** The Kaplan-Meier curve of the TICS subgroups in total PDAC patients.

A schematic of the study design and model development is shown in Figure 1. Overall survival (OS) analysis of the 661 patients in the four training cohorts (TCGA-PAAD-US, ICGC-PACA-AU, ICGC-PACA-CA, and GSE62452) and the 188 patients in the two validation cohorts (GSE71729 and GSE57495) showed no significant differences (Supplementary Figure S1). The landscape of the 28 infiltrated immune cells including the adaptive immune cells and innate immune cells was generated based on the mRNA expression in each cohort, respectively. Strong relationships among the 28 immune cells were observed in the training cohorts (Figure 2A, *P* < 0.05). We conducted a prognostic analysis for the 28 immune cells in each training cohort and performed a meta-analysis to obtain these stable and pooled hazard ratio (HR) and coefficients of all 28 immune cells (Supplementary Table S2). In brief, the activated CD4 T cells and neutrophils were risk factors (all HR > 1, *P* < 0.05), while monocytes, activated B cells, macrophages, and Th17 were protective factors for the prognosis in patients with PDAC (all HR < 1, *P* < 0.05) (Figure 2B).

### Establishment of TICS Based on the Selected Prognostic Tumor-Infiltrating Immune Cells

We established TICS for each patient based on the relative abundance of the six selected tumor-infiltrating immune cells (21). TICS had a better predicting capacity than any other of the six single immune cell alone for predicting survival through the time-dependent-ROC analysis in the training cohorts (Figure 2C, all *P* < 0.05). We also found that the expression levels of the activated B cells, monocytes, macrophages, and Th17 cells were positively related to the TICS while the activated CD4 T cells and neutrophil cells showed a negative relationship with the TICS (Figure 2D, all *P* < 0.001). All the patients in the training cohorts were then divided into two subgroups based on the best cutoff value of the TICS: the high TICS subgroup (*n* = 419) and the low TICS subgroup (*n* = 242), and a significantly different prognosis was observed (log-rank test, *P* < 0.001; Figure 2E).

### Prognosis Analysis of TICS in the Training, Validation, and Pan-Cancer Cohorts

**FIGURE 3:**
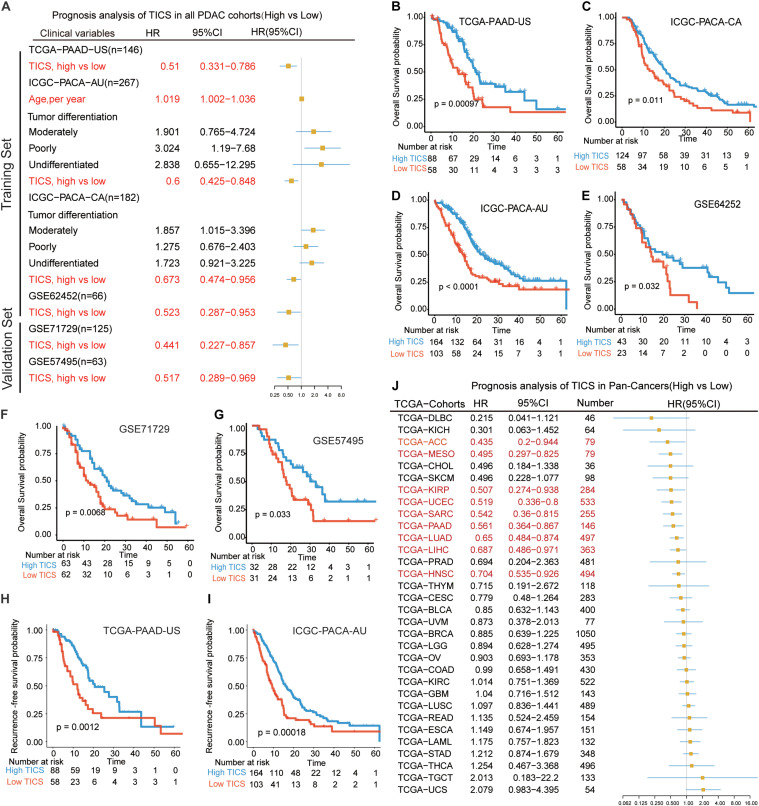
Prognostic analysis of TICS model in the training cohorts and validation cohorts. **(A)** Prognosis analysis of TICS in all PDAC cohorts (High vs. Low TICS). Kaplan–Meier survival curve of the overall survival (OS) in PDAC patients with high TICS and low TICS in TCGA-PAAD-US (*n* = 146) **(B)**, ICGC-PACA-CA (*n* = 182) **(C)**, ICGC-PACA-AU (*n* = 267) **(D)**, GSE62452 (*n* = 66) **(E)**, GSE71729 (*n* = 125) **(F)**, and GSE57495 (*n* = 63) **(G)**. Kaplan–Meier survival curve of recurrence-free survival in PDAC patients with high TICS and low TICS in TCGA-PAAD-US (*n* = 146) **(H)**, and ICGC-PACA-AU (*n* = 267) **(I)**. **(J)** Prognosis analysis of TICS in Pan-Cancers (High vs. Low TICS).

To detect the prognostic value of TICS in PDAC patients, we used the univariate and multivariate cox regression analyses to explore the risk factors for the OS of patients with PDAC in all cohorts (Supplementary Table S4). The multivariate analysis showed that only the TICS was considered as a protected prognostic factor of OS in the training cohorts (Figure 3A, *P* < 0.05). Fortunately, this conclusion also could be validated in the other two validation cohorts (Figure 3A, *P* < 0.05). According to the KM curves, we found that the TICS could easily distinguish the PDAC patients with different prognosis in all four training and two external cohorts, respectively (Figures 3B–G, *P* < 0.05). Furthermore, TICS was significantly related with the recurrence-free survival rate in the TCGA-PAAD and ICGC-PACA-AU cohorts (Figures 3H,I, *P* = 0.0012 and 0.00018, respectively). Lastly, we validated TICS for the prognosis of pan-cancers and the results showed that TICS could also significantly distinguish the prognosis of 9/32 cancers. The details of Pan-cancer analysis could be found in Figure 3J and Supplementary Table S5. To summarize, the statistically significant prognostic differences were observed in all the PDAC cohorts and some of the pan-cancer cohorts based on the TICS subgroups.

### Characteristics of Clinical Factors, Immune Molecules, and Immune Function Based on TICS

**FIGURE 4:**
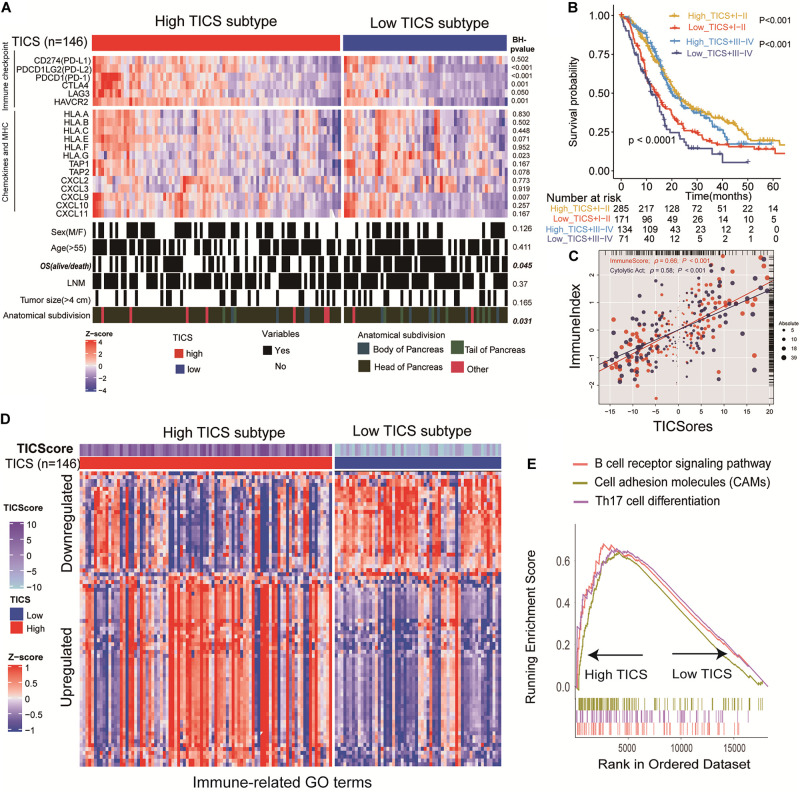
Characteristics of clinical factors, immune molecules and immune function based on TICS. This figure refers to the TCGA cohort (*n* = 146). **(A)** Specify immune checkpoints, chemokines, MHC, and clinical characteristics of the TICS subgroups. **(B)** The prognosis of the combination of TICS and TNM stages. **(C)** The correlation between the TICS and ImmuneScore/CYT. **(D)** TICS distinguishing Immune-related terms from the “msigdb.v7.0.symbols” in MSigDB; **(E)** GSEA of KEGG analysis between TICS subgroups.

The patients in the TCGA group (*n* = 146) were divided into high and low TICS subgroups. To explore the potential difference of immune status between patients in the TICS subgroups, immune checkpoints, chemokines, and major histocompatibility complex (MHC) genes were collected and analyzed based on the TCGA cohort (Figure 4A). The *P*-values of different comparisons of these representative genes between the two groups were adjusted by the method of Benjamini–Hochberg procedure. In particular, some immune checkpoint genes, such as *PDCD1LG2(PD-L2), PDCD1(PD1), CTLA4, LAG3, and HAVCR2* have a relative high expression in the high TICS subgroup than the low TICS subgroup (Figure 4A, all *P* < 0.05), except *CD274(PD-L1)* (*P* > 0.05). Conversely, among the chemokines, only *CXCL9* showed a higher expression in the high TICS subgroup (Figure 4A, *P* = 0.007). For the clinical characteristics, there is no significant correlation between the TICS subgroup and clinical parameters in the TCGA cohort (Figure 4A). Besides, there is also no significant difference in the prognosis based on the clinical TNM stage systems (I–II vs. III–IV) (Supplementary Figure S2), however, the TICS could distinguish the prognosis of PDAC within the TNM stage I–II and III–IV (Figure 4B, both *p* < 0.001). Furthermore, the TICS cores was strongly associated to both the CYC and ImmuneScore (Figure 4C, *P* < 0.001). The strong relationship between the TICS and CYC/ImmuneScore in other patient cohorts is demonstrated in the Supplementary Figure S3 (all *P* < 0.001).

After analyzing 96 immune-related gene sets for the patients in the TCGA cohort, 76 of them showed significant differences between the TICS subgroups. Among them, 51 out of 76 immune-related gene sets were up-regulated and 25 out of 76 immune-related genes were down-regulated in the high TICS group (Supplementary Table S6 and Figure 4D). It indicated that our TICS was a prognostic score which significantly related to the immune status of patients with PDAC. Besides, immune-related pathways of the B cell receptor signaling pathway, cell adhesion molecules, and Th17 cell differentiation have a higher enrichment in high TICS patients in the TCGA cohort (Figure 4E). Other results of immune-related GO and KEGG terms are shown in Supplementary Tables S7, S8.

### PDAC Somatic Genome Characteristics of the TICS Subgroups

**FIGURE 5:**
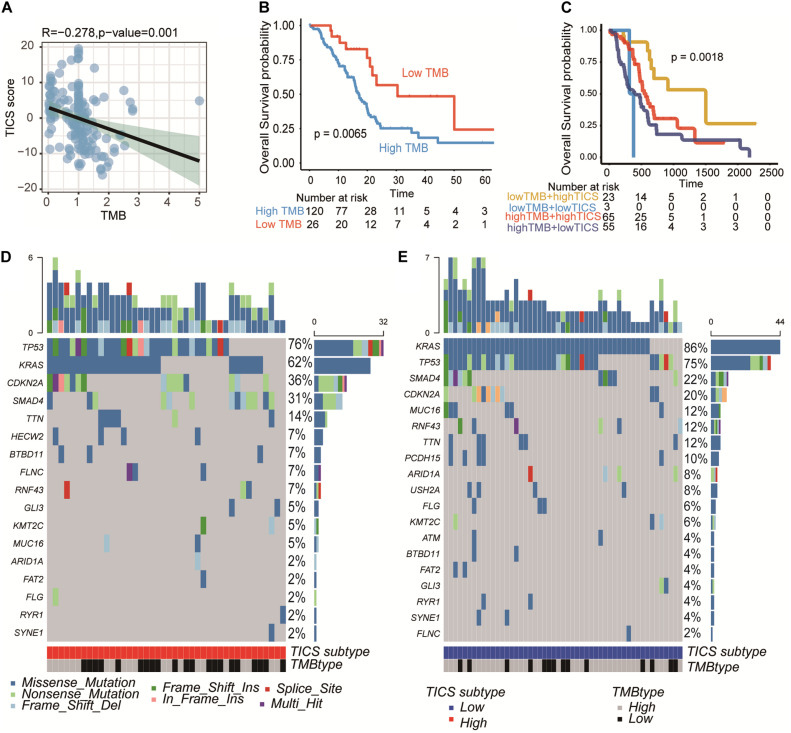
PDAC somatic genome characteristics of TICS subgroups. This figure refers to the TCGA cohort (*n* = 146). **(A)** Correlation of TICS and Tumor mutation burden (TMB). **(B)** Prognosis analysis between the low TMB and high TMB patients. **(C)** The prognosis analysis of the combination of TICS and TMB subgroups. **(D,E)** The difference of most frequent mutations genes in high TICS and low TICS subgroups, respectively.

The somatic mutations of the TCGA patients (*n* = 115) were integrated for further TMB analysis. The unique TMB score can indicate a low prevalence of somatic mutations. First, the relationship between the TICS and TMB was explored here. The scatter plot showed that TICS was negatively related to TMB scores (Figure 5A, *r* = −0.278, *P* = 0.001). Besides, patients with a high TMB showed a statistically worse overall survival (Figure 5B, *p* < 0.05). The same negative association could also be found in ICGC-PACA-AU (Supplementary Figure S4, *P* < 0.001). Notably, the combination of the TMB scores and TICS improved the prediction of the PDAC prognosis. The low TMB and high TICS groups showed the best prognosis compared with the other groups while the high TMB and low TICS groups had the worst prognosis (Figure 5C, *p* < 0.05). The difference in the mutated genes were also studied between the high TICS and low TICS groups (Figures 5D,E). Of note, the percentage of *KRAS* in the high TICS cohort were lower than that in the low TICS cohort (chi-square test, *P* < 0.05). In particular, a significant difference between the TMB genes among the different TICS cohorts was observed.

### Utilization of TICS for Chemosensitivity Prediction

**FIGURE 6:**
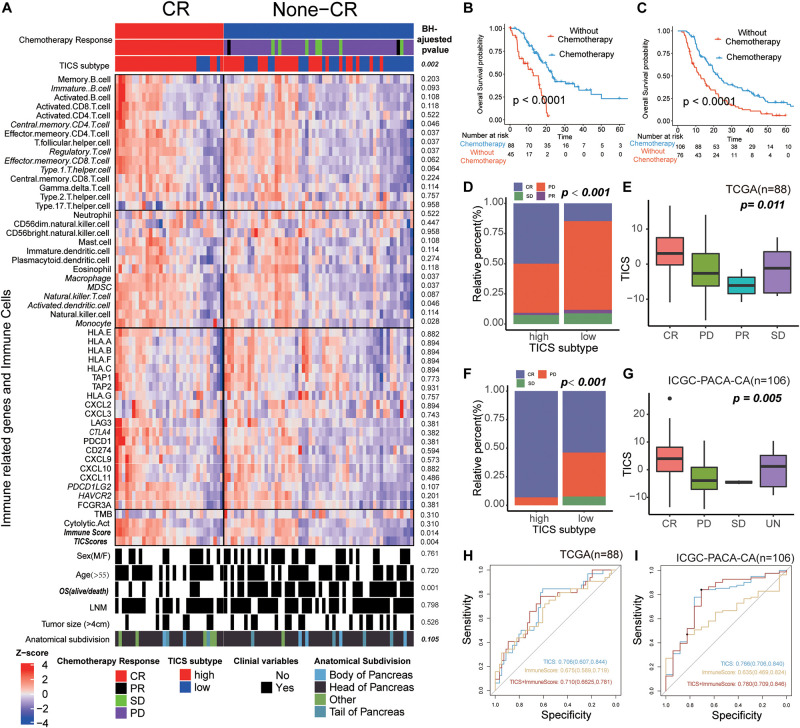
The Utilization of TICS for Chemosensitivity Prediction. These figures refer to the TCGA (*n* = 88) and ICGC-PACA-CA cohorts (*n* = 106). **(A)** Heatmap of the immune cells and immune related genes between the patients with status CR and non-CR after chemotherapy. **(B,C)** Survival analysis for patients with and without chemotherapy in TCGA and ICGC cohorts, respectively. **(D,E)** Bar and box plot for the relationship between TICS and chemosensitivity in the TCGA cohort. Complete Response (CR), Partial Response (PR), Stable Disease (SD) and Progressive Disease (PD). **(F,G)** Bar and box plot for the relationship between TICS and chemosensitivity in the ICGC-PACA-CA cohort. **(H,I)** ROC analysis of TICS and the combination with other immune-related genes for predicting the complete response status in both cohorts.

On the basis of the clinical information in the TCGA and ICGC-PACA-CA cohorts, we found that 88 and 106 patients with matching the information of chemotherapy, respectively. First, the immune cells, immune-related genes, and clinical characteristics were compared between patients with a complete response (CR) and non-CR status in the two abovementioned cohorts. The results demonstrated that only TICS (*P* = 0.004) and ImmuneScore (*P* = 0.014) showed significant differences between the CR and non-CR groups in TCGA. Furthermore, patients with CR showed a higher tendency in the immune-related genes and immune cells, although, only some of them showed statistically significance (Figure 6A). Similar results were also documented in the ICGC-PACA-CA cohort and it was even more obvious that the CR patients have a higher abundance of immune cells and immune gene expression (Supplementary Figure S5). Chemotherapy significantly improved the prognosis of patients with PDAC compared with those without chemotherapy (Figures 6B,C, log-rank test, *P* < 0.001).

Intriguing, TICS could significantly distinguish the prognosis of the PDAC patients with chemotherapy, but not in the patients without chemotherapy (Supplementary Figure S6). Besides, we found 50% and 90% patients in the high TICS subgroup with a CR status in the TCGA and ICGC-PACA-CA cohorts, respectively; meanwhile, the low TICS group showed a much smaller percentage of CR patients in both cohorts (Figure 6D, TCGA, *P* = 0.002; Figure 6F, ICGC-PACA-CA, *P* < 0.001). Besides, patients with CR had the highest TICS both in the TCGA and ICGC-PACA-CA cohorts (Figure 6E, *P* = 0.011 and Figure 6G, *P* = 0.005). However, TMB was not significantly related to the different chemotherapy responses in both cohorts (Supplementary Figure S7). We combined TICS with ImmuneScore to predict the CR status of patients with PDAC who received chemotherapy. The combined scores achieved the best prediction for CR in both cohorts compared with these two scores alone (Figure 6H, TCGA, AUC = 0.710; Figure 6I, ICGC-PACA-CA, AUC = 0.780).

## Discussion

Pancreatic ductal adenocarcinoma is a highly devastating cancer with a particularly poor prognosis. Late and inefficient diagnosis and aggressive cancer biology largely determine the unfavorable outcomes of PDAC. Adjuvant systemic chemotherapy works as the first-line postoperative treatment to strive for a long-term survival (22). Thus, a highly accurate prognostic assessment and chemotherapy response prediction of PDAC are crucial for individualized surveillance plans and clinical decisions. Therefore, this study aimed to identify a particular immune cell pattern based on the biological features of individual tumors to better categorize patients into different prognostic subgroups and identify patients who could reach a greater benefit from the adjuvant chemotherapy. At present, some other immune indicators such as ESTIMATE score (19) and Golon’s ImmuneScore (23) mainly focus on the description of suppressive immune environment characteristics. We know that even though the immune microenvironment of each tumor is similar, its prognosis is not completely consistent with the state of the immune environment. Methods such as TIMER and CIBERSORT are based on the relative expression of cells obtained by analyzing the data expression of the reference queue. The heterogeneity of the tumor caused the reference cohort to not completely conform the actual patient situation but the ssGSEA method provides a stable production route without such shortcomings. TICS has a good generalization ability for multiple PDAC tumors, which is an advantage other scores do not have. Besides, TICS integrates OS and immune microenvironment characteristics and corrects the prognostic coefficient of OS for the equivalent expression of tumor-infiltrating immune cells.

In the present study, we developed a novel immune score that predicts the survival and chemosensitivity of PDAC. Among these cells that significantly associated with the prognosis in our study, neutrophils and CD4^+^ T cells are negatively correlated with the TICS. It is widely accepted that the majority of pancreatic cancers arise from the context of chronic inflammation (21). Neutrophil and CD4^+^ cells, as the major inflammatory cells, might increase the risk of carcinogenesis and tumor progression of pancreatic cancer. This belief is fueled, in part, by the fact that the peripheral elevated tumor-infiltrating neutrophils in PDAC patients predict worse clinical outcomes (24). Of note, the CD8^+^ T cells does not included in the TICS, although the infiltrating cytotoxic T cells are associated with the efficacy of the immunotherapy, the fate of these effector cells is largely dependent on the TME (25). The immunosuppressive tumor microenvironment of PDAC might exhaust the infiltrating CD8^+^ T cells (26). In light of this point, it is important to characterize the immune subsets which might shape the pro-tumor and anti-tumor effects of the PDAC microenvironment. Nevertheless, the analysis revealed that the number of monocytes and macrophages in the tumor tissue associates with a better overall survival. It contrasts with the view that the tumor-associated macrophages suppress the immune response in PDAC. But the impact of macrophages on tumor progression cannot be easily determined as the macrophages have two faces inside the tumor: M1: anti-tumor and M2: pro-tumor. Recently, Weissman’s study has revealed that the macrophage might phagocyte the tumor cells as the innate immune cells (27). The dual roles of macrophages warrant further investigation in the PDAC. Similarly, the Th17 cells also work as double-edged swords in pancreatic cancers. Several lines of evidence have shown that the Th17 cells promote the cytotoxic T cell activation by improving the dendritic cells’ presentation of tumor antigens. Besides, the transplantation of Th17 T cell in the murine model of pancreatic cancer has the anti-tumor effect and increases the survival (28). The above findings elucidate the influence of immune cells in the improved PDAC prognosis.

Furthermore, the immune molecules, tumor, and clinical characteristics of the high and low TICS groups were also compared. The results showed many differences in immune characteristics but no significant difference in the clinical indicators. GSEA of KEGG analysis further reported differences in the immune signaling pathways between the high and low TICS patients. Therefore, the TICS staging system demonstrated a high superiority in classifying patients with PDAC in terms of the tumor immune biology. Besides, TMB has emerged as a promising novel biomarker in predicting the prognosis and immune response in various cancers (29). We discovered that high mutation rates were associated with genes (*KARS and TP53*) (30), which were mostly related to cancer progress, tumor angiogenesis, and metastasis (31–33). TICS showed a modest negative relationship with the TMB score (*r* = −0.28, *p* < 0.05). It is reasonable given the relative high immune cells contributing to the TICS value and result in low TMB. Besides, it also indicates our mRNA–based immune cell signatures could also reflect the tumor DNA somatic mutations. Although the TMB score could also distinguish patients with PDAC into a different prognosis, TICS was superior to TMB and could further stratify patients with the similar TMB scores into different subgroups.

In addition, tumor-infiltrating immune cells in the complex TME were used to predict the chemotherapy response. There were more patients with a CR status in the high TICS subgroup than in the low TICS subgroup. Patients with a CR status had a relatively higher TICS indicating the preexisting stronger immune ability of the patients achieved CR after chemotherapy. More intriguing, for patients with chemotherapy, it is documented that the high TICS patients achieved a statistically significant survival than the low TICS patients but not for patients without chemotherapy. This could be explained that chemotherapy can enhance the tumor antigen presentation by upregulating the expression of the tumor antigens themselves (34). Besides, the chemotherapy could also induce the breakdown of the cancer cells to increase the level of the tumor-associated antigen available to the antigen-presenting cells (35). In conclusion, these results revealed that the immune cell signature could help to predict the chemotherapy response.

Although our study could add more benefits for predicting the prognosis and chemotherapy of PDAC, it still has some limitations. First, we examined the cellular gene expression of 28 common immune cells that appear commonly in the TME but other types of immune cells within TME should also be included in future research. Second, both cancer cells and stromal cells orchestrate tumor-associated inflammation, tumor progression, and treatment response. Therefore, for a more comprehensive analysis of the TME, future research should include more immune cells and consider stromal cells in the TME.

## Conclusion

In conclusion, our findings revealed that the tumor-infiltrating immune cell signature can stratify patients with PDAC into subgroups with different survival outcomes. These signatures could also be used for identifying the patients that might achieve a greater benefit from the adjuvant chemotherapy. The TICS might be applied as an adjunct individualized surveillance tool to support the TNM prognostic staging system in terms of cancer immunology.

## Data Availability Statement

All datasets presented in this study are included in the article/Supplementary Material.

## Ethics Statement

All the patients’ data involved in this study is open source which is freely available in the public research databases including the Cancer Genome Atlas (TCGA), the International Cancer Genome Consortium (ICGC), and Gene Expression Omnibus (GEO). The application of the public data is already properly anonymized and informed consent was also obtained at the time of the original data collection.

## Author Contributions

YG, SC, SV, and XZ conceived and designed the project. YG and SC studied the concept and detailed design, draft preparation, data collecting, analysis, interpretation, and critical revision of the manuscript for important intellectual content. XZ and SV had supervised the project and contributed to writing and revision of the manuscript. All authors have read and approved the final revision of the manuscript.

## Conflict of Interest

The authors declare that the research was conducted in the absence of any commercial or financial relationships that could be construed as a potential conflict of interest.

## Footnotes

1 https://xenabrowser.net/datapages/
