# Supplementary material for: Tumor-Infiltrating Immune Cell Signature Predicts the Prognosis and Chemosensitivity of Patients With Pancreatic Ductal Adenocarcinoma
Source: Front Oncol. 2020 Sep 25;10:557638. doi: 10.3389/fonc.2020.557638 (PMC7545319; doi:10.3389/fonc.2020.557638)
Supplement: Supplementary file 2 [file Data_Sheet_2.docx]

**Supplementary material**

**Tumor-Infiltrating Immune Cell Signature Predicts the Prognosis and**

**Chemosensitivity of Patients With Pancreatic Ductal Adenocarcinoma**

Yuzhen Gao^1,2*^, Shipeng Chen^2*^, Somayeh Vafaei^3#^, Xiaoli Zhong^1#^

1. Department of Molecular Diagnosis, Clinical Medical College, Yangzhou University, Yangzhou, 225000, PR China;

2. Department of Laboratory Medicine, Shanghai Eastern Hepatobiliary Surgery Hospital, Shanghai, 200438, PR China

3. Department of Molecular Medicine, Faculty of Advanced Technologies in Medicine, Iran University of Medical Sciences, Tehran, Iran.

*Yuzhen Gao and Shipeng Chen contributed equally to this work and share co-first authorship.

^#^Corresponding author:

Xiaoli Zhong^#^, Department of Molecular Diagnosis, Clinical Medical College, Yangzhou University, Yangzhou, PR China. E-mail: [xlzhong@yzu.edu.cn](mailto:xlzhong@yzu.edu.cn)

Somayeh Vafaei^#^, Department of Molecular Medicine, Faculty of Advanced Technologies in Medicine, Iran University of Medical Sciences, Tehran, Iran. Email: Somayeh.vafaei@gmail.com.

**Contents**

[**Figure S1. The overall survival (OS) of all patients in 4 training cohorts and 2 validation cohorts.** 3](#_Toc50671273)

[**Figure S2. The prognostic value of TNM stage in the training cohorts with available clinical TNM information.** 4](#_Toc50671274)

[**Figure S3. The relationship of TICS with CYC and Immune score in the training cohorts and validation cohorts.** 5](#_Toc50671275)

[**Figure S4. The relationship of TMB with TICS in ICGC-PACA-AU and CA cohorts.** 6](#_Toc50671276)

[**Figure S5. The relationship of Chemotherapy with other variables in the ICGC-PACA-CA cohort.** 7](#_Toc50671277)

[**Figure S6. The prognostic value of TICS in the patients with or without chemotherapy in the TCGA and ICGC-PACA-CA cohorts.** 8](#_Toc50671278)

[**Figure S7. The relationship between TMB and chemotherapy response in the pancreatic cancer.** 9](#_Toc50671279)

# **Figure S1. The overall survival (OS) of all patients in 4 training cohorts and 2 validation cohorts.**


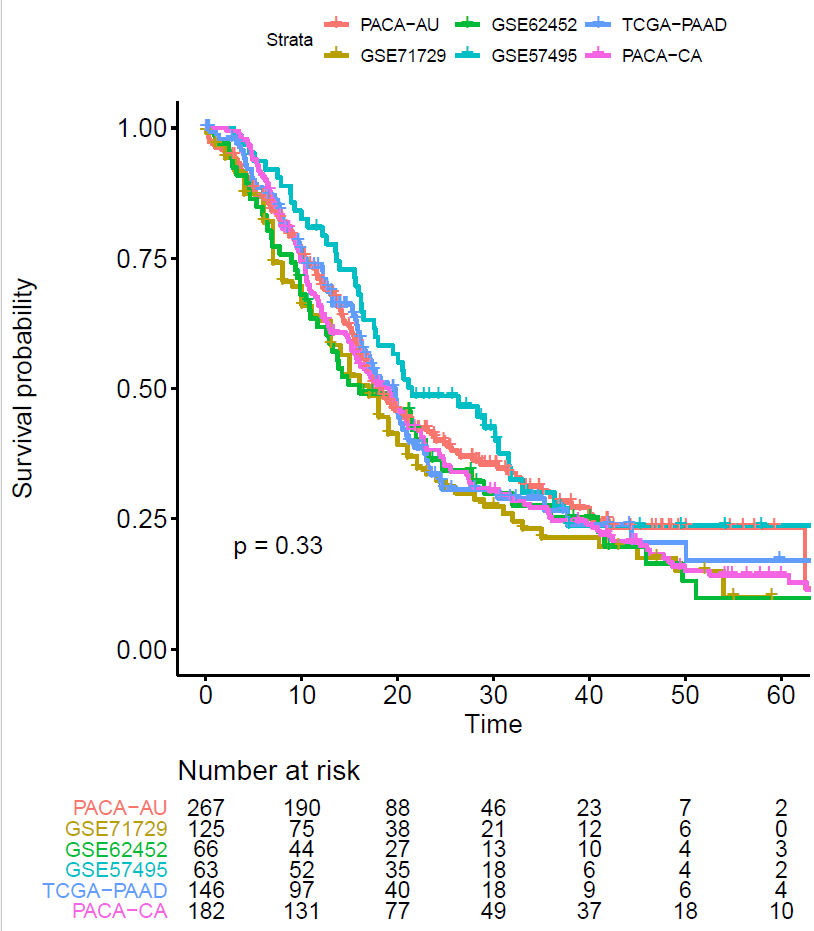


# **Figure S2. The prognostic value of TNM stage (I-II vs III-IV) in the training cohorts with available clinical TNM information.**


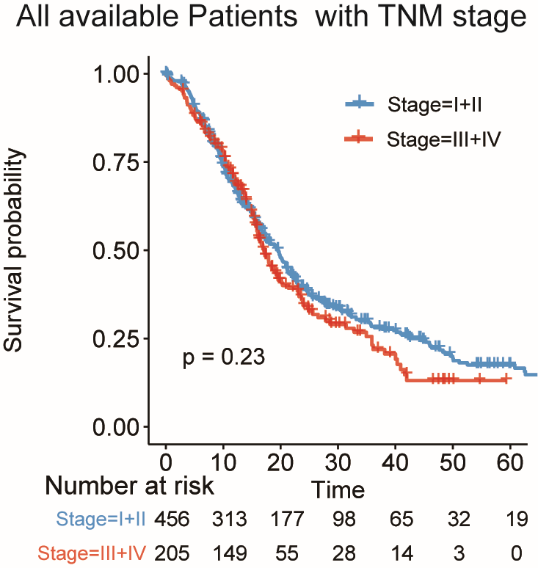


# **Figure S3. The relationship of TICS with CYC and Immune score in the training cohorts and validation cohorts.**

**A:**
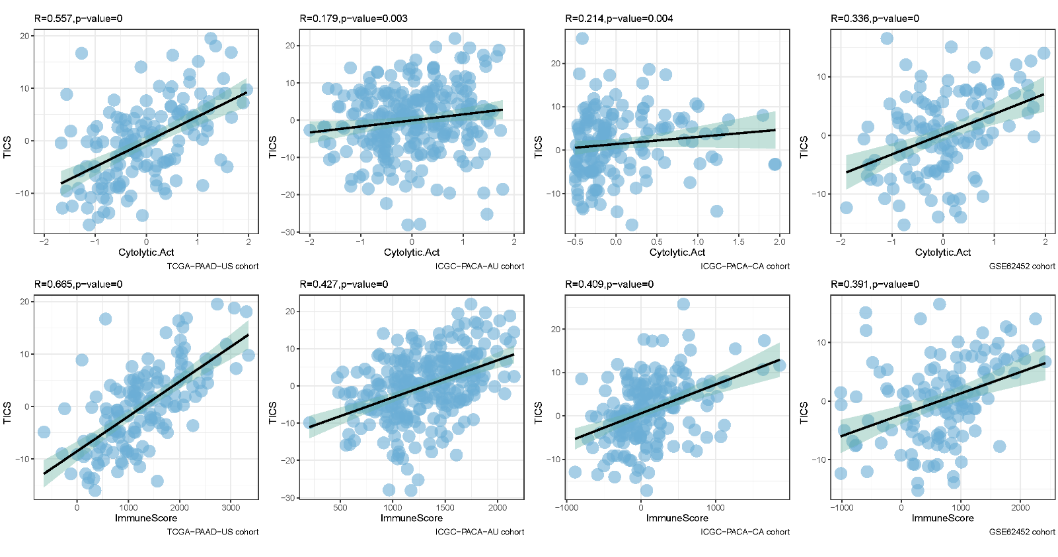


**B:**
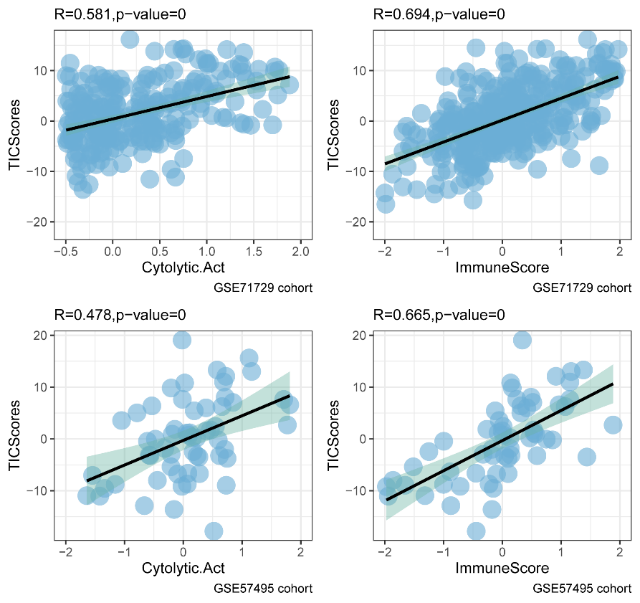


(A) The four training cohorts (TCGA-PAAD-US, ICGC-PACA-CA, ICGC-PACA-AU, GSE64252).

(B) Two validation cohorts (GSE57495 and GSE71729).

# **Figure S4. The relationship of TMB with TICS in ICGC-PACA-AU and CA cohorts.**


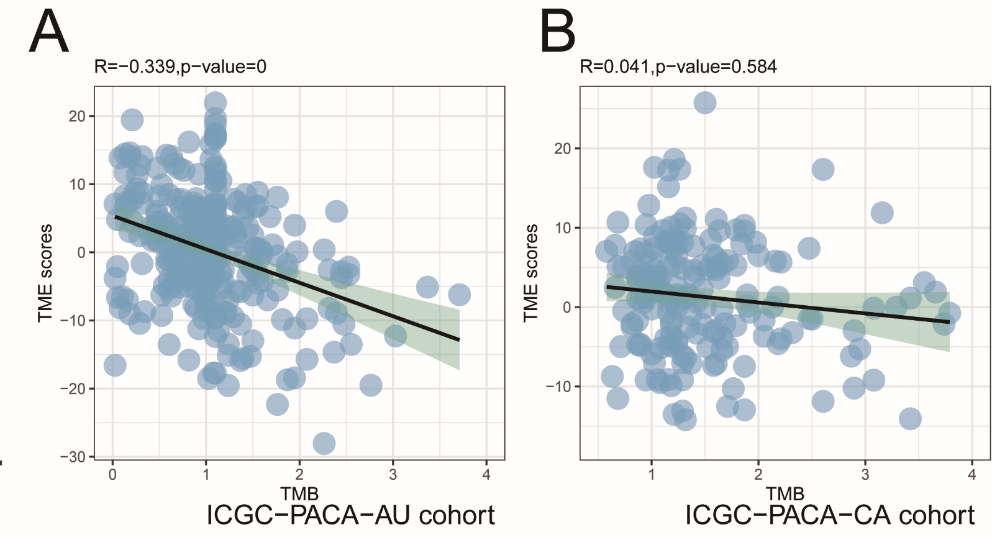


**(**A) The relationship of TMB with TICS in the ICGC-PACA-AU.

(B) The relationship of TMB with TICS in the ICGC-PACA-CA.

# **Figure S5. The relationship of Chemotherapy with other variables in the ICGC-PACA-CA cohort.**


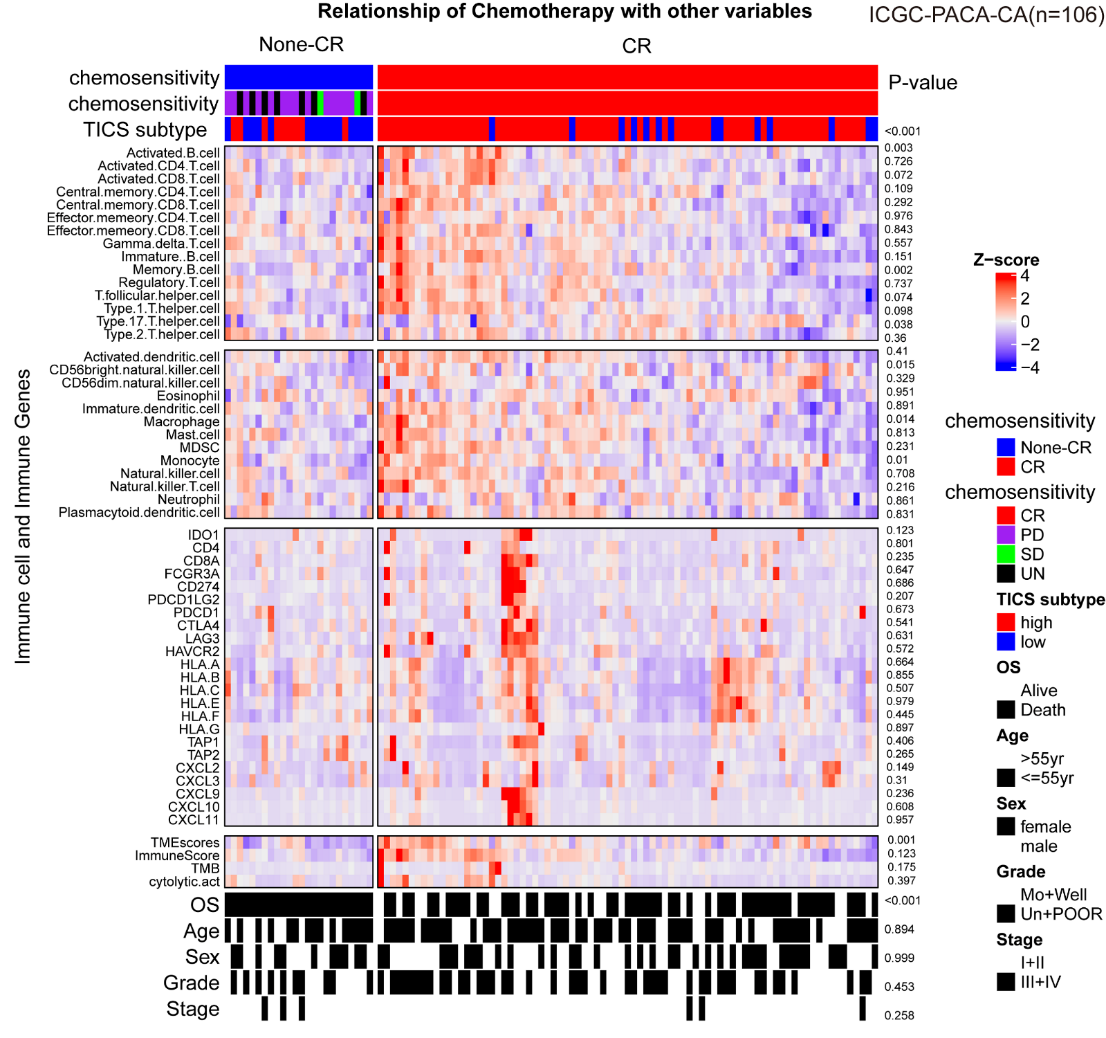


The heatmap showed the differences of representative immune related genes, 28 immune cells and clinical variables in the patients with chemotherapy from ICGC-PACA-CA cohorts.

# **Figure S6. The prognostic value of TICS in the patients with or without chemotherapy in the TCGA and ICGC-PACA-CA cohorts.**


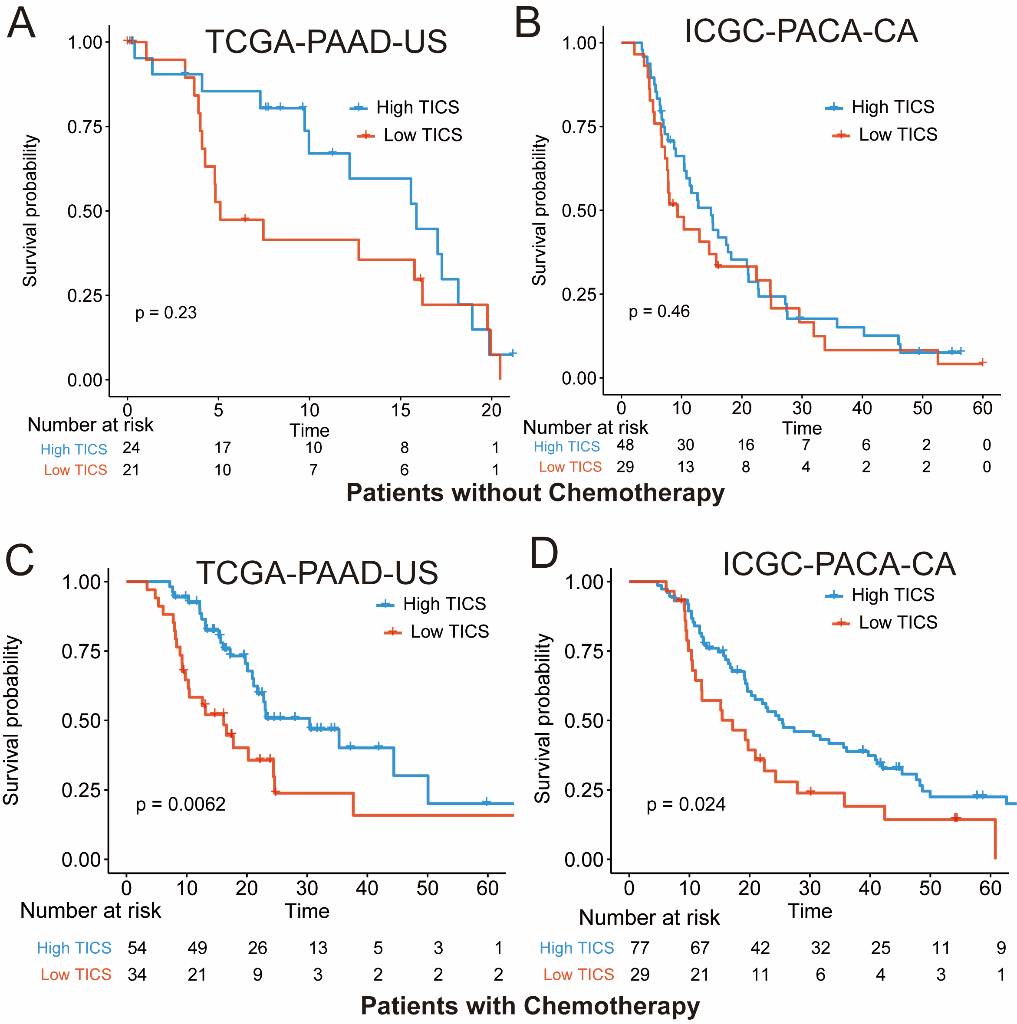


(A, B) The KM-curves of high and low TICS subgroups in the patients without chemotherapy from TCGA-PAAD-US and ICGC-PACA-CA cohorts, respectively.

(C, D) The KM-curves of high and low TICS subgroups in the patients with chemotherapy from TCGA-PAAD-US and ICGC-PACA-CA cohorts, respectively.

# **Figure S7. The relationship between TMB and chemotherapy response in the pancreatic cancer.**


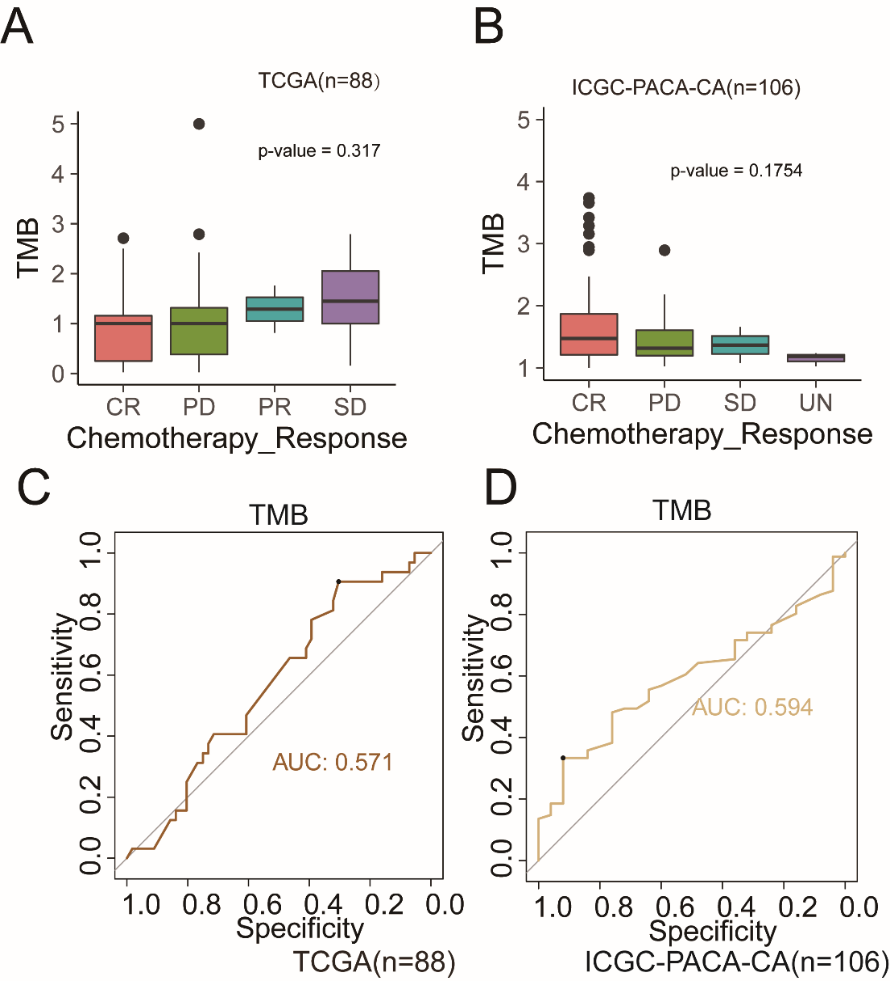


(A)The difference of TMB among different chemotherapy response in the patients with PADC in the TCGA-PAAD-US cohort (n=88). (B) The difference of TMB among different chemotherapy response in the patients with PADC in the ICGC-PACA-CA cohort (n=106). (C) The predictive value of TMB for predicting the complete response (CR) of the patients with PADC and chemotherapy in the TCGA-PAAD cohort. (D) The predictive value of TMB for predicting the CR of the patients with PADC and chemotherapy in the ICGC-PACA-CA cohort;
